# DNA-Based Single-Molecule Electronics: From Concept to Function

**DOI:** 10.3390/jfb9010008

**Published:** 2018-01-17

**Authors:** Kun Wang

**Affiliations:** Department of Mechanical Engineering, University of Michigan, Ann Arbor, MI 48109, USA; kunwang@umich.edu; Tel.: +1-734-936-2331

**Keywords:** DNA, molecular electronics, charge transport, molecular junctions, single-molecule conductance, *I–V* characteristics

## Abstract

Beyond being the repository of genetic information, DNA is playing an increasingly important role as a building block for molecular electronics. Its inherent structural and molecular recognition properties render it a leading candidate for molecular electronics applications. The structural stability, diversity and programmability of DNA provide overwhelming freedom for the design and fabrication of molecular-scale devices. In the past two decades DNA has therefore attracted inordinate amounts of attention in molecular electronics. This review gives a brief survey of recent experimental progress in DNA-based single-molecule electronics with special focus on single-molecule conductance and *I–V* characteristics of individual DNA molecules. Existing challenges and exciting future opportunities are also discussed.

## 1. Introduction

Building an electronic device out of single molecules represents the ultimate miniaturization of active electronic components. Not only could such molecular-scale devices conceivably increase circuit density by thousands of folds compared to today’s state of the art silicon-based technologies, but the unique geometrical properties of molecules also add overwhelming freedom and flexibility for the design of functions not possible with conventional semiconductors. Since the initial proposal of this attractive idea by Aviram and Ratner in 1974 [1], a large body of research has been stimulated to probe charge transport (CT) through individual molecules both theoretically and experimentally [2,3,4,5,6,7,8,9,10,11]. Thanks to rapid development of experimental techniques in recent years [3,4,12,13], researchers are now able to reliably and repeatedly wire an individual molecule between two metallic electrodes and study their CT properties. Recent experimental advances include the demonstration of conductance switching [11,14,15,16], rectification [9,17,18,19], negative differential conductance [20,21], and other promising phenomena beyond simple electron transport, such as quantum interference [22,23,24], thermoelectricity [25,26], optoelectronics [27,28,29], spintronics [30,31] and Peltier cooling [32]. 

**Figure 1:**
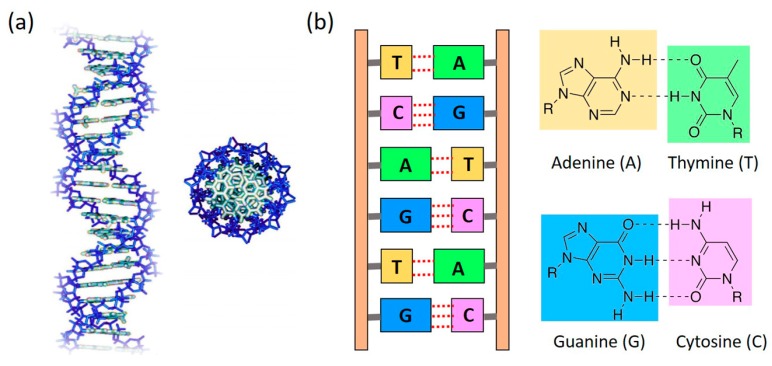
(**a**) Schematic representation of a double helix DNA from side view (**left**) and top view (**right**); (**b**) DNA base pair (bp) coupling between Adenine (A)—Thymine (T) via two hydrogen bonds (dotted lines) and Guanine (G)—Cytosine (C) via three hydrogen bonds (dotted lines).

**Figure 2:**
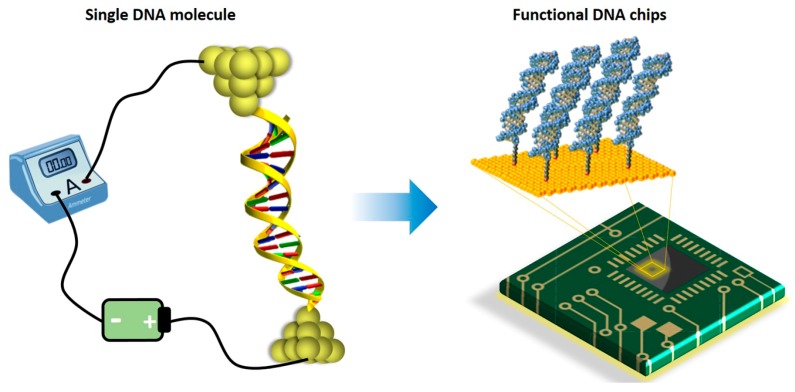
Vision from DNA single-molecule junction to future DNA-based molecular chips.

DNA, known as the reservoir for genetic information, has recently caught enormous attention in the field of molecular electronics. This is mainly because the overlapping electronic orbitals of the stacked DNA bases together with the ability to program both sequence and length in a nearly endless number of combinations make DNA an excellent one-dimensional system for CT studies (Figure 1). In addition, DNA’s ability to form error-free self-assembled nanostructures, such as DNA origami [33,34,35], with no need for expensive microfabrication technologies renders it a leading candidate to be exploited as a template for assembling nanoscale integrated circuits. In the meantime, probing CT through DNA offers opportunity to answer questions associated with biological processes, such as oxidative damage of DNA [36,37]. These unique properties of DNA hold huge potential for the development and application of future real-life molecular electronic devices and biosensors (Figure 2), and it has therefore attracted many researchers, applying a variety of techniques to DNA CT studies, in the past two decades. 

Investigation of the possibility of electric conduction in DNA was first raised by Eley and Spivery in 1962 [38]. In the following years, Barton and coworkers [39] pioneered electron transfer through DNA using photoelectron transfer systems where electron acceptor and donor molecules were introduced into a short DNA sequence. In their studies, CT through DNA were measured by injecting electrons from one transition metal intercalated site along a DNA duplex chain and collecting the outputting current from another site that is a few base pairs (bps) away. Details about this method can be found in several excellent reviews (see refs. [39,40,41,42]). However, understanding charge transport through a structure where an individual DNA molecule is wired to two metallic electrodes represents the most fundamental step towards achieving the goal of building DNA-based molecular circuits. The experimental attempts to measure the electrical conductance of such nanoscopic structure started from the 1990s. However, confusion emerged in the early days. Due to poor control and understanding of experimental conditions, the experimental results were all over the map. A wide range of CT properties of DNA have been reported, including semiconductor [7], superconductor [43], and insulator [44,45] by different groups using different experimental approaches. Most of these results were perhaps mainly due to different measurement conditions and methods which made precise interpretation of DNA’s conformation difficult and sometimes even induce contaminations [40]. Thanks to recent development of single-molecule break junction techniques and other detailed measurements, concensus has been reached that a short DNA molecule is a one-dimensional conductor and can be used as molecular wires since the frontier orbitals of the bases, the highest occupied molecular orbital (HOMO) and the lowest unoccupied molecular orbital (LUMO), are π-electrons residing orbitals perpendicular to the molecular plane [40,46,47]. 

Substantial progress has been made to fabricate DNA-based single-molecule junctions and characterize their CT properties in the past two decades. In this review, we aim to provide an overview of recent experimental advances in probing charge transport through various DNA molecules. Special focus will be given to single-molecule conductance and *I–V* characteristics of DNA. We also introduce works that probed other interesting physical properties of DNA, such as piezoelectricity, thermoelectricity and spintronics. Potential future directions and outstanding challenges will also be discussed.

## 2. Experimental Approaches towards DNA Single-Molecule Conductance

**Figure 3:**
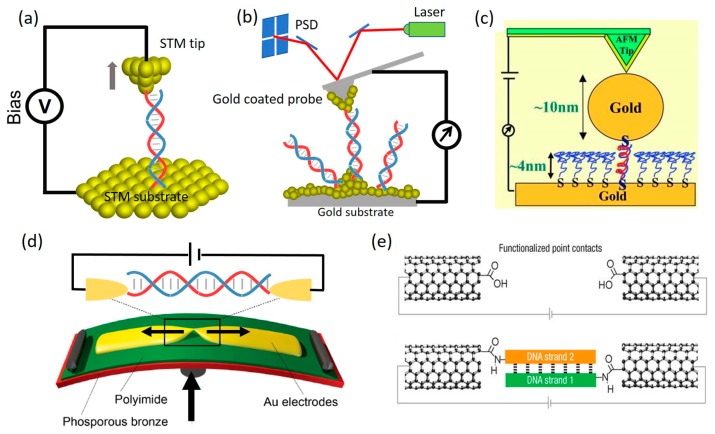
Single-molecule break-junction techniques (SMBJs). (**a**) Scanning tunneling microscope break-junction (STM-BJ) technique; (**b**) Conductive probe atomic force microscope break-junction (CPAFM-BJ) technique; (**c**) CPAFM-BJ involving nanoparticle capped DNA molecule. Reprinted with permission from ref. [51]. Copyright (2005) National Academy of Sciences, USA; (**d**) Mechanically controlled break-junction (MCBJ) technique; (**e**) SWCNT-molecule-SWCNT molecular junction. Reprinted with permission from ref. [13]. Copyright (2008) from Nature publishing group.

To determine the electrical conductance of an individual molecule such as DNA, it is necessary to wire the target molecule to two probing electrodes in a reliable and repeatable manner. However, this had remained very challenging until the introduction of scanning probe microscopy (SPM) techniques, i.e., scanning tunneling microscopy (STM) and atomic force microscopy (AFM), into the field of molecular electronics. SPM techniques have been therefore regarded as the major driving force for molecular electronics because it has made great contribution to the fabrication and modulation of single-molecular junctions and will continue to promote advances of molecular electronics in the future. In 2003, Xu and Tao [4] demonstrated the STM break junction (STM-BJ) method which for the first time enabled repeatable formation of metal-molecule-metal junctions in a very reliable fashion (Figure 3a). In this method, a metal STM tip, the movement of which is precisely controlled by a piezoelectric transducer, is repeatedly driven in and out of contact with the substrate electrode adsorbed with sample molecules. The molecules have chance to form metal-molecule-metal junctions by bridging both the tip and the substrate electrodes when the tip is brought close enough to the substrate. The tip is then withdrawn away from the substrate to break the metal-molecule-metal junctions. By accurately controlling the movement of the tip, the number of bridged molecules can be changed and a single molecule junction can form during the tip retraction process. By collecting thousands of conductance vs. tip displacement traces during tip retraction, a conductance histogram can be then constructed to reveal well-defined peaks at integer multiples of a fundamental conductance value. This histogram-based method enables one to identify the conductance of a single molecule. Nowadays, this STM-BJ technique has turned out to be the most widely-used experimental platform to measure the conductance of various single molecules, including DNA [48,49,50].

As an alternative, the conductive probe AFM break junction (CPAFM-BJ) method, a technique closely related the STM-BJ, uses a metal-coated AFM tip as the tip electrode and involves a laser-reflection controlled force signal detector, which enables measurements of conductance and force in parallel (Figure 3b) [52,53]. Unlike STM-BJ which uses current to control tip-positioning, CPAFM-BJ uses force to control tip-positioning. While a similar conductance histogram can be constructed to determine single-molecule conductance, a corresponding force histogram can also be plotted to identify the force features associated with the formation and rupture of molecular junctions. Besides, by parking a metal-coated AFM tip on top of a self-assembled monolayer (SAM) of target molecules controlled with a setpoint contact force, CPAFM method allows one to measure current through a monolayer of hundreds of molecules [12,54,55]. In addition, with the capability to image surface profile of the sample, STM- and AFM-based techniques are also used to target molecules that are custom-modified on the substrate surface, for instance, nanoparticle capped DNA molecules [51] as illustrated in Figure 3c. This can be achieved by imaging the sample surface prior to the electrical measurements.

Another method to construct metal-molecule-metal junctions is called mechanically controlled break-junction (MCBJ) technique. This method was first used by Reed et al. [3] to measure the properties of single molecules in 1997. MCBJ uses notched metal wire fixed onto an elastic substrate [56,57]. A diagram of the MCBJ is shown in Figure 3d. The substrate is usually covered with an insulator, and the metal wire is mechanically broken by bending the substrate with a pushing rod from the bottom side of the substrate. A single-molecule junction is formed when only one molecule is left in the gap between the two terminals of the broken metal wire. This method has been widely-adopted in molecular electronics because it allows one to build a three-terminal molecular transistor by introducing a gate electrode into the two-terminal metal-molecule-metal system [25,50,57]. 

Instead of using metal electrodes, carbon-based electrode materials, such as single-walled carbon nanotube (SWCNT) [11,13,58] and graphene [59,60], have also been exploited to sandwich single molecules via creating nanoscale gap and chemically connecting target molecules to carbon electrodes. Guo et al. [13] demonstrated the first measurement of charge transport through a SWCNT-DNA-SWCNT junction in 2008 (Figure 3e). In their work, a nanogap with a controllable size can be fabricated through a combination of the electrical breakdown and the electron beam-induced decomposition (EBID). A wide gap was firstly formed by passing a high-density current through a SWCNT followed by exposure to organic vapor. Then an electron beam with an appropriate energy was used to induce the EBID to regulate the gap size of the CNTs. To bind with CNT electrodes, DNA was modified with amino groups at the two terminals to form an amido group with the carboxylic group on the gapped CNTs.

The above-mentioned techniques for creating metal-molecule-metal junctions have been extensively adopted to probe CT properties of individual DNA molecules, including single-molecule conductance and *I–V* characteristics of DNA. These techniques have been reviewed in several outstanding review papers [49,50,56,61,62], and will not be revisited in detail here. 

## 3. Charge Transport through Native DNA Molecules

CT through native DNA has been the main stream of experimental investigations in DNA-based molecular electronics in the past two decades. This section gives an overview of some of the most representative experimental works that have advanced our understanding of DNA CT and paved pathways for future studies.

### 3.1. Environmental Effect: Wet vs. Dry

The experimental discrepancies in DNA conductivity measurements [7,45,46] at the intersection of the century were thorny enough to trigger additional investigations of the experimental conditions where DNA conductivity was measured. The variances in the experimental results formed the basis of numerous theoretical calculations which aimed at answering questions such as what is the role of water molecules and counter ions surrounding DNA strands [40,63,64,65]. In 2000, Tran et al. [45] measured the contactless ac conductance of λ-DNA in a buffer solution, which showed that the conductance of the DNA in solution was an order of magnitude higher than the conductance in the dry state. This, in principle, is consistent with multiple theoretical predictions [63,64] which suggested that DNA has better base pair (bp) stacking in wet conditions as it maintains B-DNA compared to that in dry conditions where it is in A-DNA form. In addition, it was also predicted that positive protons or metal counterions are necessary to neutralize and stabilize DNA. Water plays a crucial role as the hydrophobic forces make DNA form a double helix, and the polarity of the water molecules helps screen DNA’s charges [63]. It has been suggested that better base-pair stacking and coupling and stable DNA conformation are key to DNA conductivity and it is preferable for them to be formed and maintained in aqueous conditions. To meet the quest for high conductance of DNA, later experiments were therefore mostly performed in aqueous solutions containing metal counter ions.

### 3.2. Single Strand and Mismatched DNA

**Figure 4:**
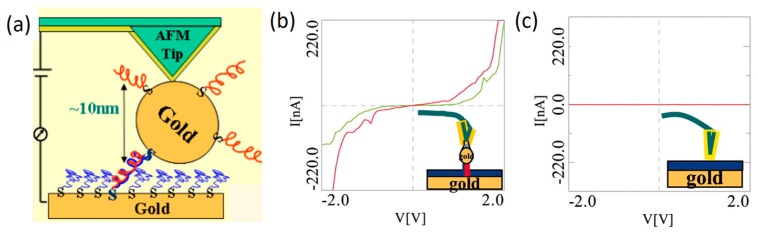
(**a**) Schematic of CPAFM-based method with a nanoparticle capped DNA molecule sandwiched between AFM tip and substrate; (**b**) Measured *I–V* curves of double strand DNA was performed on a metal particle without pressing on it; (**c**) *I–V* curve measured on the ssDNA monolayer without pressing it. Negligible current was observed here, indicating that the ssDNA monolayer is insulating. Reprinted with permission from ref. [51]. Copyright (2005) National Academy of Science, USA.

Single strand DNA: many DNA molecules studied in the field of molecular electronics consist of two self-complementary single strand (ss) DNA. It is, therefore, important to first understand CT through ssDNA because it helps to not only interrogate the possibility of using ssDNA as a molecular wire but also understand the potential effect of uncoupled ssDNA on CT measurements focusing on double strand (ds) DNA. In 2005, Cohen et al. [51] studied charge transport through a monolayer formed by both double strand (ds) DNA and single strand (ss) DNA using CPAFM method (Figure 4). In their work, it was found that negligible current was observed for ssDNA monolayer, which indicated that single strand DNA monolayer is nearly insulating as it lacks the π-electron stacking structure of ds DNA which serves as the major pathway for charge migration. It was later confirmed in other STM-BJ measurements that ssDNA has a conductance several orders of magnitude lower than that of the corresponding dsDNA [9]. Similar results were reported using STM I(t) method [66]. Nowadays, ssDNA is usually considered as an insulator and detrimental to building molecular circuits despite its versatile functions in facilitating DNA self-assembled nanostructures.

**Figure 5:**
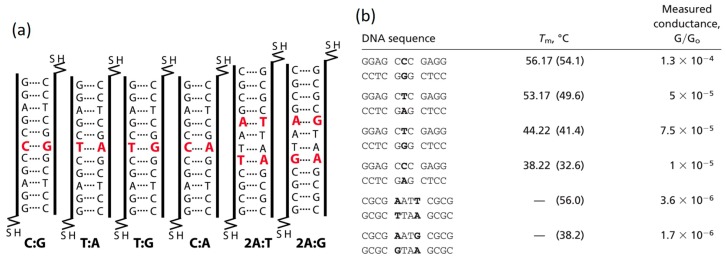
Effect of mismatched base pair on DNA conductance. (**a**) Mismatched DNA sequences studied in the work by Hihath et al.; (**b**) Measured single-molecule conductance of DNA molecules presented in (**a**). Reprinted with permission from ref. [69]. Copyright (2005) National Academy of Science, USA.

Mismatched DNA: Base pair (bp) mismatch can occur in dsDNA as the consequence of errors in replication or mutations in base-stacks, which have been considered the major cause of genetic diseases such as cancer [67,68]. While biologists invest tremendous efforts to repair mismatched bps, experimentalists in molecular electronics consider it a crucial step to study the influence of bp mismatch on the conductance of DNA before real use of DNA as a molecular wire. In 2005, Hihath et al. [69] measured the conductance of a series of dsDNA molecules with different type of mismatched bps using STM-BJ technique, as shown in Figure 5a. The conductance measurement results (Figure 5b) showed that the alteration of a single base in the stack can either increase or decrease the conductivity of the dsDNA helix, depending on the type of the mismatched. For example, while switching a T-A bp to T-G bp only increased the conductance of the DNA by 50%, changing a well-matched C-G bp to C-A can decrease its conductance by over an order of magnitude. Both the electronic states of each base and the structural instability due to a mutated base were suggested to be responsible for the conductance change in the molecule. It should be noted that the conductance change reported in this work may vary if the sequence and length of the studied DNA are altered. However, the quantifiable change in conductance of the molecule caused by a single-base mutation offer opportunity to electronically identify bp mutation in DNA.

Given both ssDNA and mismatched bp in dsDNA could potentially reduce the conductance of DNA dramatically and introduce complexity into the system, research efforts in recent years have been more concentrated on well-matched DNA molecules with different sequences and bp modifications.

### 3.3. Sequence- and Length-Dependent CT through DNA

Charge transport through double helical DNA and related charge transfer processes have received increasing interest over recent decades because of its intriguing transport properties that differentiate DNA from other molecules studied in the field of molecular electronics. For most molecules, when wired between metal electrodes, their CT is governed by either coherent tunneling, where the conductance of the molecule decreases exponentially with molecular length, or incoherent hopping, where the resistance increases linearly with length [70]. However, CT through DNA has shown strong length- and sequence-dependent transport phenomena where coherent tunneling [42,47], incoherent hopping [42,47,71] and intermediate hopping-tunneling [72] could all be the dominant transport regimes depending on the specific sequence chosen. Catalyzed by this unique trait of DNA, numerous experimental investigations have been performed using different approaches. In what follows, we will discuss some of the representative experimental discoveries that shape the current understanding of CT through DNA.

**Figure 6:**
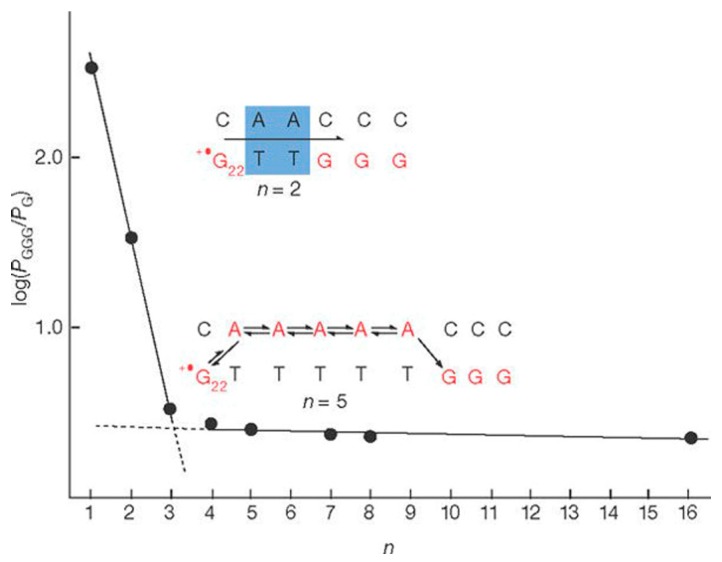
Measurement of charge transfer rate by Giese et al.: log(*P*_GGG_/*P*_G_) against the number *n* of the A:T base pairs The steep line corresponds to the coherent tunneling charge transfer. Reprinted with permission from ref. [76]. Copyright (2001) Nature Publishing Group.

Although it was indeed supported by many photoinduced charge transfer measurements [73,74,75] in the early 2000s that long range transport through DNA was mediated by multistep hopping reaction involving positive charges (holes) moving between guanines (Gs), the DNA bases with lowest ionization energy, detailed experimental investigation yielded exceptions when specific DNA sequence was chosen. In 2001, Giese et al. [76] experimentally studied the rate of charge transfer through G bases of DNA, separated by adenine-thymine (A:T)*n* bridges of various lengths, in double strands. As shown in Figure 6, they found that the rate of charge transfer between two guanine bases decreases exponentially with increasing separation only if the guanines are separated by no more than three (A:T) base pairs; if more bridging base pairs are present, the transfer rates exhibit only a weak distance dependence. This distinct change in the distance dependence of the rate of charge transfer through DNA to a shift from coherent superexchange charge transfer (tunneling) at short distances was attributed to a process mediated by thermally induced hopping of charges between adenine bases (A-hopping) at long distances. This work highlighted that a change in charge transfer mechanism can occur in a distance-dependent fashion.

**Figure 7:**
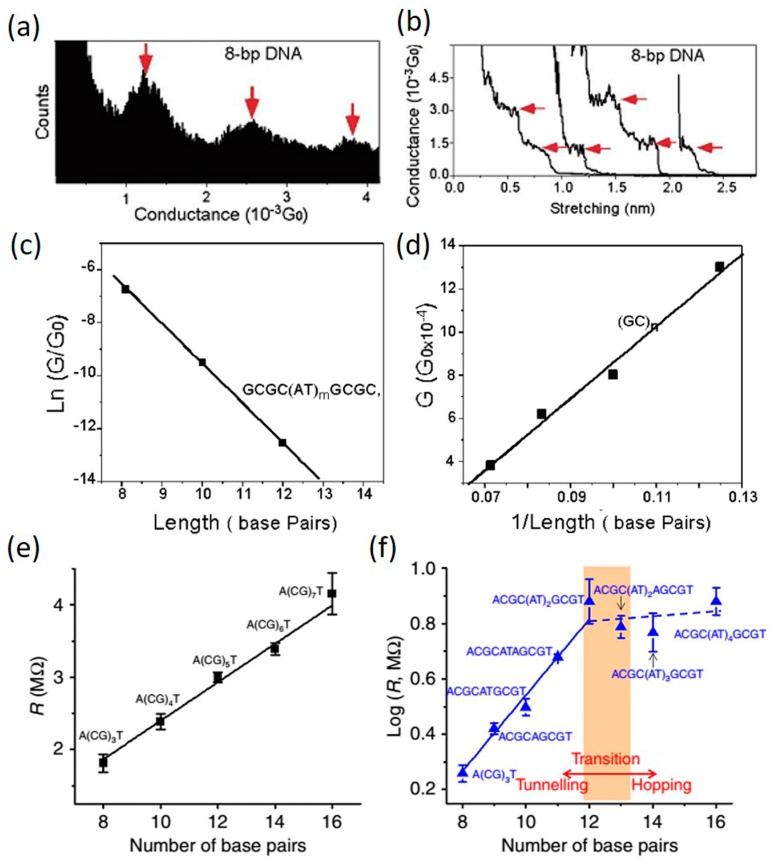
Sequence and length dependent conductance (G) of single DNA molecules. Panel (**a**,**b**) show the example conductance histogram and conductance vs. distance traces, respectively; (**c**) ln(G/G_0_) vs. molecular length plot for DNA with ACGC(AT)*_m_*GCGT sequences; (**d**) G vs. 1/length plot for DNA with (GC)*_n_* sequences; (**e**) Resistance of A(CG)*_n_*T DNA as a function of number of base pairs; (**f**) Log R vs. number of base pair plot for DNA with ACGC(AT)*_m_*GCGT/ACGC(AT)*_m_*AGCGT sequences. (**a**–**d**) are reprinted with permission from ref. [47]. Copyright (2004) American Chemical Society. (**e**–**f**) are reprinted with permission from ref. [77]. Copyright (2016) Nature Publishing Group.

To gain deeper insight into the results obtained in the previous charge transfer measurements, researchers also interrogated the single-molecule conductance of various DNA sequences and lengths using the emerging STM-BJ technique. In 2004, Xu et al. [47] measured the single-molecule conductance of a series of dsDNA molecules containing different number of alternating (AT)*_m_* in the middle of alternating (GC)*_n_* bps in aqueous solutions. The experimental results are shown in Figure 7a–d. They found that for (GC)*_n_* sequences, the conductance is inversely proportional to the length (greater than eight base pairs) (Figure 7d). Contradictory to previous findings where short range transport through molecules was governed by tunneling [42], the conductance of short DNAs with (GC)*_n_* sequences decreases very slowly with the length. When inserting (A:T)*_m_* into GC-rich domains, it decreases exponentially with the length of A:T base pairs with a decay constant of 0.43 Å^−1^ (Figure 7c). It was highlighted that this decay constant of the studied DNAs is much lower than alkanes and peptides, indicating that DNA is much more conductive than alkanes and peptides. This work demonstrated different conduction mechanisms for different sequences even when the DNA is short. The results reported in this work were later confirmed in other measurements. Li et al. [77] recently reported conductance measurements of DNA molecules with similar sequences but longer length. In their work, the resistance (inverse of conductance) of DNAs with A(CG)*_n_*T sequences showed a linear length-dependence behavior (Figure 7e), consistent with the results in Xu’s work. In contrast, the resistance length-dependence of ACGC(AT)*_m_*GCGT/ACGC(AT)*_m_*AGCGT is significantly different. For *m* = 0, 1 and 2, the resistance increases more rapidly with length and can be best fitted with an exponential function. Within this range of molecular length, the results agree well with those in Xu’s work. For *m* > 2, however, the resistance becomes weakly length dependent, indicating a transition in the charge transport mechanism. This observation further verified the results reported in the DNA charge transfer rate measurements by Giese et al. (Figure 6). To briefly summarize at this point, it has been accepted now that the conduction mechanism of native dsDNA is dominated by tunneling (exponential dependence of conductance on length) when the G-C bps are separated by three or fewer AT bps. If the number of A-T bps is increased, diffusive hopping (linear dependence of conductance on length) becomes the main transport mechanism. However, for (GC)*_n_* sequences, the conductance of DNA is usually governed by thermally activated hopping with G bases as the hopping sites.

**Figure 8:**
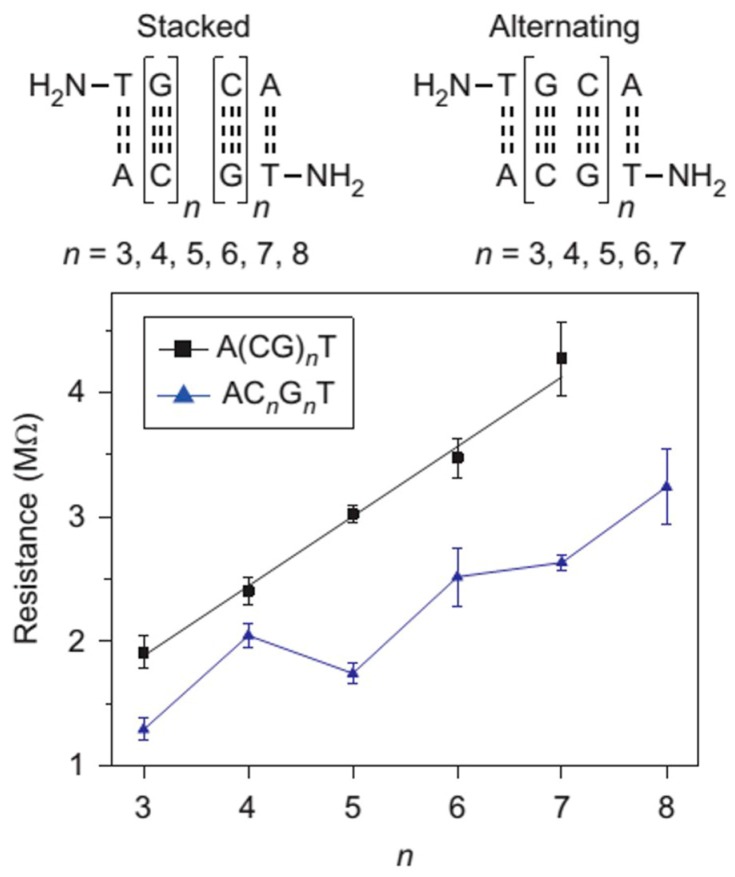
Single-molecule conductance measurements of DNA with alternating G (A(CG)*_n_*T) and stacked G (AC*_n_*G*_n_*T) sequences. Reprinted with permission from ref. [72]. Copyright (2015) Nature Publishing Group.

However, it was recently found that the hopping model no longer held when the alternating G is changed to stacked G sequences. This was reported in a recent study by Xiang et al. [72] In their work, single-molecule conductance of DNA duplexes with alternating G and stacked G sequences in different lengths were measured using STM-BJ method (Figure 8). It was found that in good agreement with previous results, the resistance of alternating G (black dot in Figure 8) showed a linear growth with the increase of molecular length and the results can be described with the hopping model. In contrast, the resistance of the stacked G sequences showed a surprising periodic oscillation superimposed on the linear length dependence. With the assist of theoretical simulations based on Buttiker theory, this oscillation phenomenon is attributed to a partially coherent and partially hopping regime of charge transport. The calculations revealed that the HOMOs in the stacked G are delocalized over several G bases, supporting the observation of an intermediate coherent tunneling and incoherent hopping charge transport mechanism. A more detailed follow-up theoretical investigation [78] further suggested that coherence dominates charge transport for odd-n sequences, whereas incoherent hopping dominates even-n sequences. The CT in odd-length sequences cannot be dominated by incoherent transport because polaron formation in incoherent CT will stabilize the polaron states and remove the mid-gap levels, which would destroy the energy distinction between odd- and even-length sequences. As such, resistance oscillations would disappear. When *n* ≥5, the block delocalization is expected to be disrupted by thermal fluctuations, oscillations are expected to be weak and the resistance is expected to become Ohmic. 

### 3.4. Structure-Dependent Transport in DNA

DNA molecules have proven to exhibit surprising conformational versatility, while retaining remarkable precision and uniformity [79]. It can adopt different conformations in solution depending on the sequence and environment. The best characterized form of DNA is the right-handed, B-form, helix, which is normally adopted by dsDNA in physiological conditions [80]. We note that the conformation of DNA molecules described earlier in the present review is in B-form. DNA can also adopt other structures, including a right-handed but more compact helix known as the A-form and a left-handed helix known as Z-form. It has been reported while the presence of ethanol in addition to alkaline metal ions in solution may cause a right-handed internal switch from B- to A-DNA [81,82], increasing the concentration of alkaline metal ions in the solution could shift the right-handed B-DNA to a left-handed Z-DNA [83,84,85]. 

Apart from the fact that the conformational transition of DNA from one to another is of biological and medical significance [86,87,88], understanding its influence in CT properties has remained a central question in molecular electronics as it has been suggested as one of the underlying reasons for the large variation in early DNA conductance measurements. Recently, researchers explored both the influence of DNA’s structural transition from B- to Z-form and from B- to A-form on its conductance using STM-BJ method.

**Figure 9:**
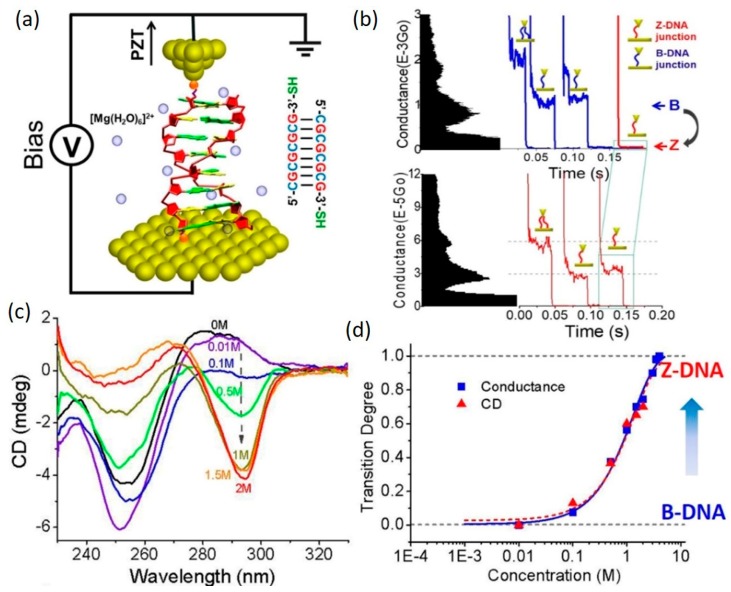
Conductance measurements of poly(GC)_4_ DNA under a structural transition from B- to Z-form. (**a**) Schematic of STM-BJ method; (**b**) Example conductance histograms and traces of B-DNA (upper) and Z-DNA (lower) in 1 M MgCl_2_ solution; (**c**) CD spectra measured in 0–2 M MgCl_2_ solutions; (**d**) Transition degree vs. log MgCl_2_ concentration plot, showing the entire transition process monitored by conductance measurement. Reprinted with permission from ref. [79]. Copyright (2014) Royal Society of Chemistry.

B-Z transition: using STM-BJ method, Wang et al. [79] studied the conductance change of a 8 bp poly(GC)_4_ DNA duplex induced by a structural transition from B- to Z-form (Figure 9a). In their work, the B- to Z-DNA transition was facilitated by increasing the concentration of Mg^2+^ ions in the buffer solution from 0 M to 4 M. The structural change was monitored and confirmed by circular dichlorism (CD) measurements (Figure 9c). Through measuring the single-molecule conductance of DNA, they found that the conductance of DNA molecule decreased by two orders of magnitude as DNA conformation transfers from B- to Z-form (Figure 9b). The cause of this conductance reduction is primarily attributed to the structural change-induced breaking of π-π orbital stacking between neighboring bps which may come from a few sources, including the rise between adjacent bps by 14%, axial angle twist from 36° in B-DNA to −30° in Z-DNA and flipping of G bases by almost 180°, which have a detrimental effect on distribution of effective orbitals for CT. Therefore, DNA in Z-form is not suggested for molecular electronics applications. Interestingly, this work also showed that when three A-T bps were bridged in the middle of the (GC)_4_ sequence, such B-Z structural transition effect was not present and therefore no significant change in DNA conductance was observed even when Mg^2+^ concentration in the solution increased from 0 M to 4 M. It was also highlighted that the B-Z transition can be monitored simply by measuring the conductance of the DNAs even when traditional method such as CD fails (Figure 9d).

**Figure 10:**
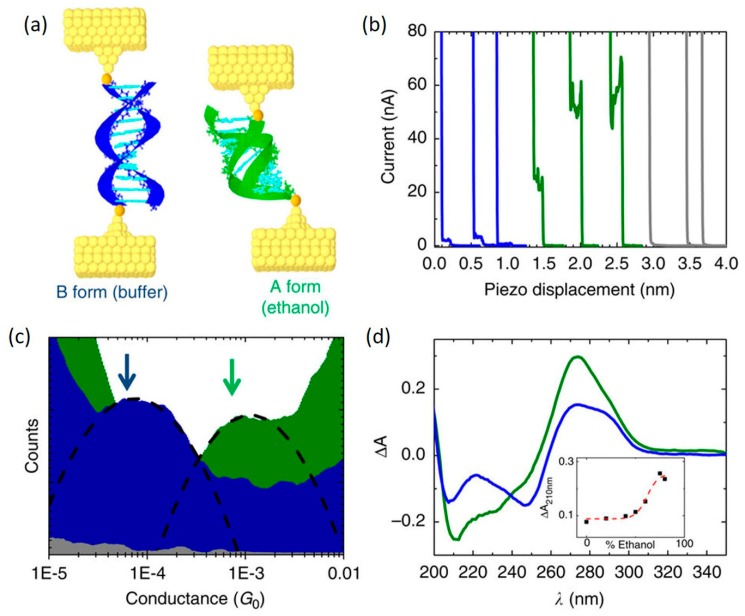
Conductance measurements of DNA under B-A structural transition (**a**) Schematic of B- and A-DNA molecular junction; (**b**) Example conductance traces of B-DNA (blue) and A-DNA (green); (**c**) Example conductance histograms of B-DNA (blue) and A-DNA (green); (**d**) CD spectra of B-DNA (blue) and A-DNA (green) sample. Reprinted with permission from ref. [89]. Copyright (2015) Nature Publishing Group.

B-A transition: change in conductance of DNA induced by a B-A structural transition was experimentally explored by Artes et al. [89] It should be noted that before this work the base alignment in A-form was expected to be worse than that in B-form, which led to the prediction that A-form is insulating [63]. However, the results of this work showed that the conductance of DNA duplexes with a GC rich sequence of 5′-CCCGCGCGCCC-3′ increases by approximately one order of magnitude when its conformation changed from B-form to A-form (Figure 10). The B- to A-form transition was achieved by changing the solution from phosphate buffer to 80% ethanol. This conductance increase by one order of magnitude was also observed in DNA molecules with longer lengths. They also found that this large conductance increase is fully reversible, and by controlling the environment, the conductance can be repeatedly switched between the two values. However, length-dependent conductance studies of the two conformations suggest that neither tunneling nor simple hopping dominate the charge transport processes in these guanine-rich sequences. ab initio electronic structure calculations coupled with calculations of the electronic density of states of the two conformations revealed that the HOMO, the dominant transport orbital, is distributed over 70% of molecular length in the A-form, but only 50% in the B-form and that the A-form transmission function is orders higher than B-form transmission, therefore leading to a higher conductance of A-form DNA. By directly demonstrating the conformational change plays a significant role in the transport properties, the results of these studies also help rationalize the large dispersion of DNA conductivity found in the literature.

**Figure 11:**
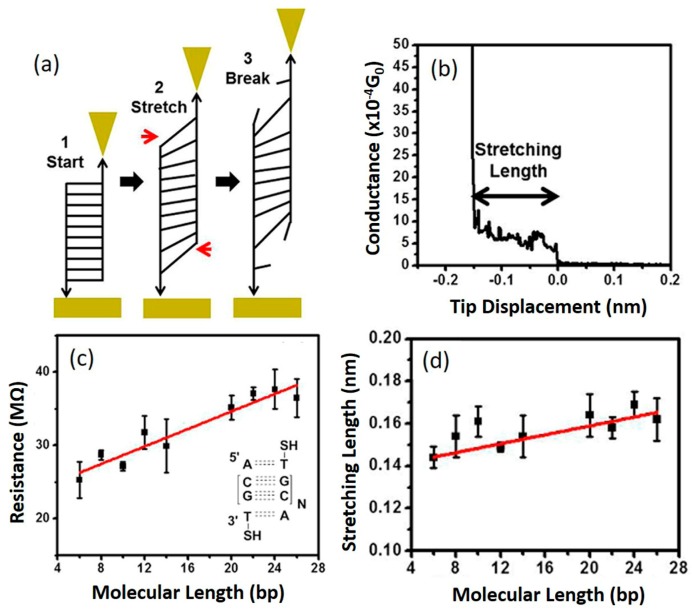
Stretching effect of DNA conductance. (**a**) Illustration of the evolution of dsDNA duplex during stretching; (**b**) Example conductance trace for dsDNA sequence 5′-S(CG)_2_T-3′; (**c**) Resistance vs. molecular length plot; (**d**) Stretching length vs. molecular length plot. Reprinted with permission from ref. [98]. Copyright (2015) American Chemical Society.

Effect of stretching: one important structural perturbation dsDNA duplex may undergo is being stretched, leading to a dramatic structural change. Indeed, it has been shown that DNA undergoes a structural transition when mechanically stretched [90,91,92]. This has been attributed to either a reversible configuration change from native form (B-DNA) to stretched form (S-DNA) or irreversible force-induced melting [93,94,95,96,97]. As charge transport in dsDNA molecules is mediated by the π-π stacking interactions between neighboring base pairs, mechanically stretching DNA is believed to seriously disrupt the π-π stacking interactions and therefore leads to a large change in CT of DNA [98]. Bruot et al. [98] recently reported their experimental study of the effect of mechanical stretching on DNA conductance (Figure 11). Using STM-BJ method, they explored the effect of the stretching transition on dsDNA conductance by analyzing the evolution of single-molecule conductance during electrode separation. dsDNA molecules (5′-A(CG)_N_-3′, N = 2~12) with lengths varying from 6 bps (~2 nm) to 26 bps (~9 nm) were studied in aqueous environment. It was found that although the single-molecule conductance of studied DNA molecules showed linear length dependence (Figure 11c), consistent with previous results, CT of DNA is highly sensitive to mechanical stretching (Figure 11b), showing an abrupt decrease in conductance at surprisingly short stretching distances, which is weakly dependent on the molecular length (Figure 11d). These unexpected observations are attributed to a force-induced melting mechanism [99] and are consistent with de Gennes’ DNA ladder model [100] for dsDNA mechanics. Based on de Gennes’s ladder model, as shown in Figure 11a, the abrupt conductance decrease is caused by the breaking of the hydrogen bonds of the base pairs near the ends by the external shear force (~3 pN) which decrease the coupling between the dsDNA hopping sites and the electrodes. It is worth noting that Bruto et al. reported a new molecule–electrode linker based on a hairpin-like design which could overcome this force-induced melting at the end of single DNA molecules during stretching [101], opening up the possibility to construct DNA electromechanical devices.

*G4-DNA*: Guanine-quadruplex (G4) DNA, an important derivative of dsDNA, has also drawn considerable attention in molecular electronics over the past decade [102,103,104]. This is mainly because G4-DNA has several advantageous structural traits over dsDNA. First, given that short dsDNA with G-C rich sequence has been reported to be more conductive than A-T sequence [47,105], the stacking of several G-quartets in G4-DNA is predicted to yield high conductance [106]. Second, unlike the relatively flexible conformation of dsDNA, the quadruple helical conformation ensures that the G4-DNA is rather stable under physiological conditions and indicates higher stiffness and stronger resistance to surface forces than the dsDNA [102,107]. Third, G4-DNA is an excellent prototype to study self-assembling properties at the supramolecular scale and the design of biomimetic systems [107,108]. Due to these superior features, G4-DNA has been considered a promising candidate as a molecular wire.

**Figure 12:**
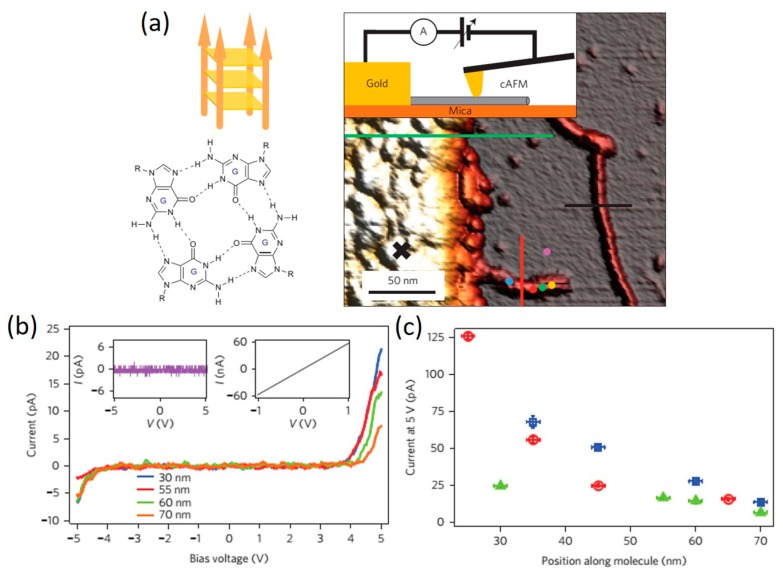
(**a**) biotin-avidin (BA)–G4-DNA scheme showing an oriented ordered stack of tetrads and AFM image showing a typical measurement scenario: a gold electrode with a sharp edge is on the left and molecules are clearly visible on the mica to the right; one molecule (at the bottom) is protruding from under the edge of the metal electrode; (**b**) *I–V* measurements taken at the positions indicated by colored dots on the molecule shown in (**a**); (**c**) Distance dependence of the current measured at a bias of 5 V for three different molecules (plotted in different colors). Reprinted with permission from ref. [102]. Copyright (2014) Nature Publishing Group.

Direct measurement of charge transport through G4-DNA has been carried out very recently. In 2014, Livshits et al. [102] reported the *I–V* measurement of a long G4-DNA using an experimental setup depicted in Figure 12a. In their work, a gold electrode was evaporated using stencil lithography on top of a long biotin-avidin (BA)–G4-DNA (~250 nm) which had been pre-adsorbed onto a flat mica surface. By contacting the molecule with a metalized AFM tip, *I–V* curves were collected at different locations along the long G4-DNA chain. Negligible current below ±3–4 V followed by a rapid rise in the current was observed in all *I–V* curves, as shown in Figure 12b. In addition, a weak and non-trivial dependence of the *I–V* data, especially the current rise, on the distance between the measured point and the border of the electrode (Figure 12c) was observed. The obvious asymmetry of the *I–V* curves was attributed to asymmetric coupling at the metal-molecule interfaces (the contact at the evaporated electrode is much better and more reproducible than the tip-molecule contact). Conducted in a very controllable manner, their experiments showed that G4-DNA can transport significant amount of current over long distances when deposited on a hard substrate, which is the more relevant configuration for solid-state devices, and the long-range transport through G4-DNA is mediated by thermally activated hopping over stacked G-tetrad blocks.

**Figure 13:**
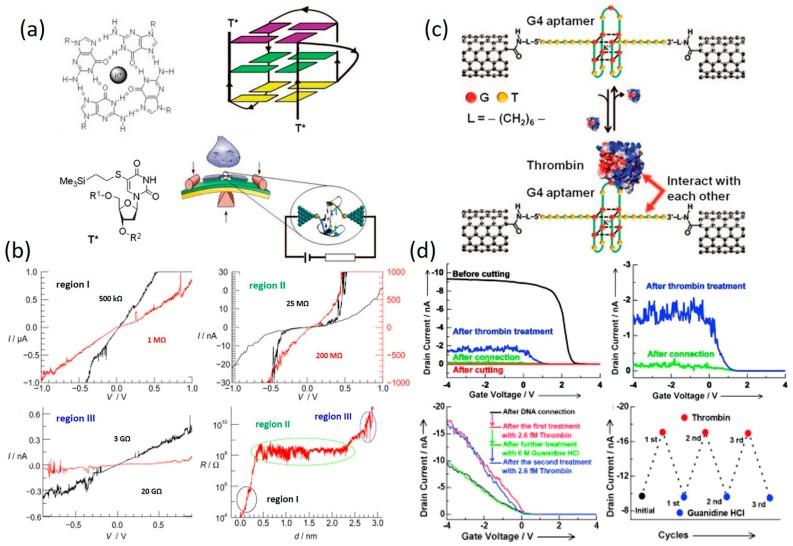
(**a**) Schematic illustration of G4-DNA measured by MCBJ setup; (**b**) *I–V* curves measured at different at different stages of G4-DNA defolding. (**a**,**b**) are reprinted with permission from ref. [104] Copyright (2010) Wiley–VCH Verlag GmbH & Co., KGaA. (**c**) Schematic representation of the CNT-G4-DNA-CNT sensing setup for protein detection; (**d**) Current vs. gate voltage curves measured at a constant source-drain voltage of −15 mV before and after thrombin treatments, showing reversible current change at two discrete levels; (**c**,**d**) are reprinted with permission from ref. [109]. Copyright (2011) Wiley–VCH Verlag GmbH & Co. KGaA.

Single-molecule conductance of short G4-DNA has also been performed recently using single-molecule break junction methods. Using the MCBJ approach, Liu et al. measured the conductance of a short G4-DNA (5′-T*G3[TTAGGG]3T*)-3′, T* is modified thymine to couple the molecule to the electrodes) which was covalently bonded to Au electrodes through thiol-Au interaction [104]. In their study, a pronounced and flat conductance plateau with a length of around 2 nm was observed (Figure 13b, bottom-right panel). It was noticed the plateau resistance remains almost constant while the G-quadruplex conformation is present. *I–V* measurements performed at the molecular plateau region showed a highly non-linear, with a typical S shaped *I–V* curve of G4-DNA. Although detailed transport mechanism was unclear, this work also showed that G4-DNA can transport considerable current at reasonable voltages in the range of 1 V. More importantly, contrast to dsDNA, the resistance of the G4-DNA molecule is quite independent of the elongation of the molecule. Liu et al. recently studied the possibility of using SWCNT-G4-DNA-SWCNT junction as a protein-detection device [109]. Their experimental setup involved connecting a 15-mer thrombin DNA aptamer with thymine 7 (T7) linkers on both the 3′ and 5′ termini to nanogapped SWCNTs, as shown in Figure 13c. Under a constant source-drain voltage of −15 mV, the current vs. gate voltage signal was constantly monitored when the G4-DNA aptamer was connected between the SWCNTs before and after thrombin treatment. It was found that the current of the SWCNT-G4-aptamer-SWCNT junction increased dramatically upon treatment of thrombin and this change is reversible over several cycles by alternating treatment with thrombin (2.6 fM) and guanidine HCl (6 M). To understand the drastic conductance change induced by G4-DNA interacting with thrombin, they hypothesized that DNA–thrombin interactions do not distort the G4 conformation, but instead rigidify the G4 conformation and promote tight packing, thus enhancing the conductance of the G4-DNA. Apart from measuring CT through G4-DNA, their work demonstrated how G4-DNA can be used to interface with biological processes for the development of bio-detection devices. 

Collectively, experimental studies discussed in this section have delivered an important message that CT through DNA is highly sensitive to the structure of DNA, and it therefore requires better understanding of the correlation between DNA’s conformation and its conductance before DNA can be used in future molecular electronic devices.

## 4. Charge Transport through Modified DNA Molecules

The ability to effectively tune the electronic structure of native DNA molecules is key to satisfying the quest for high conductance and versatile functionalities of DNA. To achieve this, experimentalists have attempted to modify native dsDNA molecules using multiple approaches, such as methylation [110,111], metallo-base pair [112], and small molecule intercalation [9,113]. In this section, various modifications used in molecular electronics experiments and their effect on CT through DNA will be discussed. 

### 4.1. DNA Methylation 

**Figure 14:**
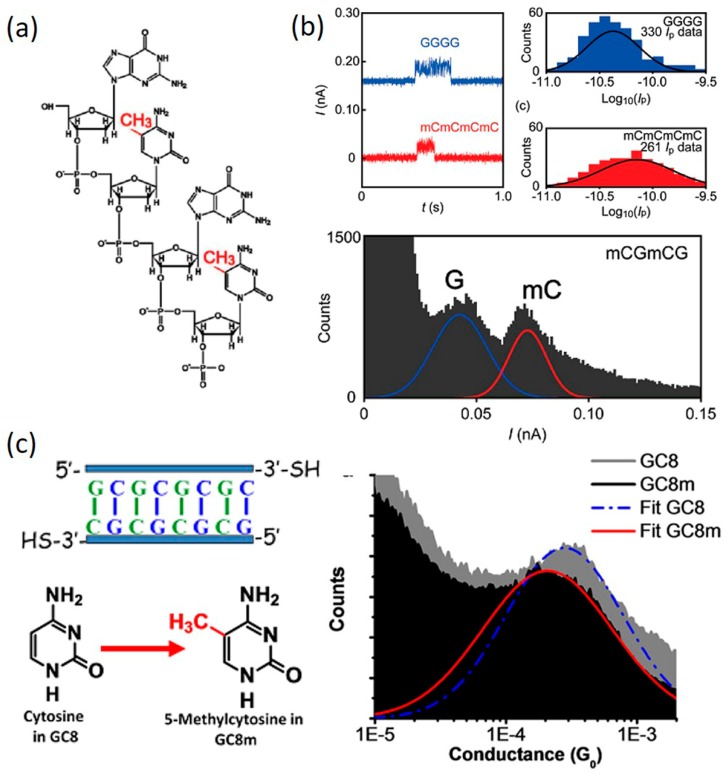
(**a**) Structure of a DNA oligomer with sequences of GGGG and mCmCmCmC; (**b**) Current signal (**upper**) and histogram (**lower**) of the DNA. (**a**,**b**) are reprinted with permission from ref. [110]. Copyright (2011) American Chemical Society (**c**) **Left**: DNA sequence and structure of cytosine and 5-methylcytosine; **Right**: conductance histograms for native DNA (grey) and methylated DNA (black). (Reprinted with permission from ref. [111]. Copyright (2012) IOP Publishing.

Among DNA modifications, DNA methylation constitutes an essential epigenetic mechanism for various important biological processes such as embryonic development, replications, and aging [110,114,115]. Beyond its significant role as a biomarker closely associated with human health and disease, DNA methylation has also been regarded as one possible method to tune the CT properties of native DNA as it is anticipated to affect the π-conjugation of the pyrimidine (or purine) bases considering the electron-donating character of methyl substitutions [110]. In 2011, Tsutsui et al. [110] reported their experimental investigation of the effects of methylation on the conductance of deoxycytidine-5′-monophosphate. The studied DNA 4-mers have sequences of 3′-mCGmCG-5′ and 3′-GGGG-5′, where cytosine bases were methylated, as shown in Figure 14a. The conductance measurements were carried out using MCBJ method under a constant bias of 0.4 V. It is worth noting that the measurements were performed transverse to a ssDNA rather than longitudinally as in most of the other papers discussed. Upon statistically collecting the electrical signal when a DNA oligomer was trapped between Au electrodes, they found that methyl substitution contributes to increase the tunneling conductance of deoxycytidines (Figure 14b). This conductance increase was attributed to a shift of the highest occupied molecular orbital level closer to the electrode Fermi level by methylation. This result also suggested a possible use of the transverse electron transport method for a methylation level analysis.

However, Hihath et al. [111] reported a different observation when studying the effect of the methylation of cytosine bases on DNA conductance. Using the STM-BJ method, they measured the conductance of a poly(GC)_4_ DNA duplex before and after methylation of its cytosine bases (Figure 14c). The measurement results showed the methylated DNA has a lower conductance than the its native counterpart even though the methylated DNA is more stable and has cytosine bases with a lower energy gap. They reasoned that since charge transport through GC rich sequence is dominated by hopping through guanine bases, methylation of cytosine bases will have little effect on the energy levels of guanine itself and it may not imply a better coupling between bases in the DNA stack. The difference between the work by Tsutsui et al. and the work by Hihath et al. could be reasonable because in Tsutsui’s work, charge transports in the lateral direction across the base pair, which is perpendicular to the axis of DNA. It is, therefore, more sensitive to the methylation. However, accurate understanding of these experimental discrepancies will require more detailed investigation of methylation status of individual cytosine bases within the stack of the specific DNA sequence. However, these studies suggested that DNA methylation has a clear impact on the CT properties of DNA.

### 4.2. Metallo-DNA

Metals are the carriers of conducting electrons which are particularly desired in the field of molecular electronics. It was therefore envisioned that doping the interior of oligonucleotide duplexes with metal ions may yield hybrid materials with enhanced conductivity or other interesting electronic effects [116]. Toward this direction, extensive research efforts have been put to both covalently attach metal complexes to native DNA and create “metal-base pair” by replacing the hydrogen-bonded Watson–Crick base pairs with metal–ligand interactions inside the DNA double helix [117]. We refer readers to other excellent reviews that cover the related topics (see refs. [116,117]). 

The possibility of using metal-DNA as electronic device was first investigated theoretically: Lee and co-workers presented a model system for a field-effect transistor based on metal-DNA [118]. They proposed that a gate voltage applied perpendicular to the helix axis will cause displacement of the metal ions (an ionic Stark effect) and induce differences in the site energies which will consequently modulate the hopping rate. However, Joseph et al. [119] studied the effect of a T–Hg–T base pair (see below) on the long-distance radical cation hopping properties but found no significant effect of this metal-base pair on the charge transport. Since then, more efforts were invested to explore the optimal conditions for metal ions to couple with natural DNA base pairs.

**Figure 15:**
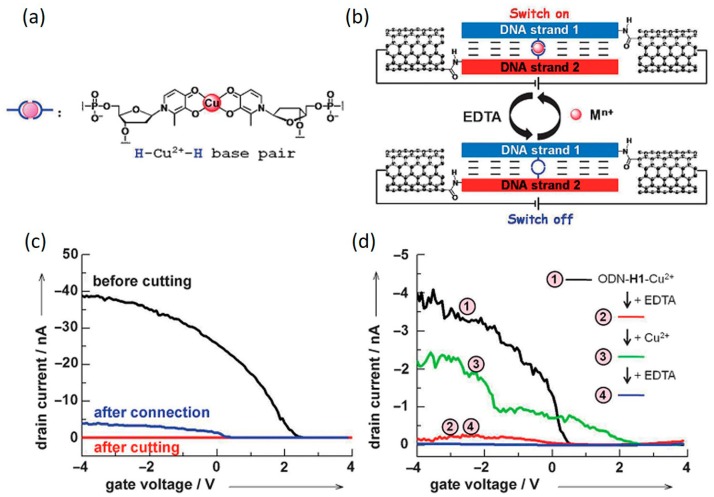
(**a**) The molecular structure of Cu^2+^ mediated base pair; (**b**) Components of metallo-DNA-bridged SWCNT devices; (**c**) Current vs. gate voltage signals of a device reconnected with metallo-DNA (blue); (**d**) Current vs. gate voltage under periodic treatment of Cu^2+^ and EDTA. Reprinted with permission from ref. [112]. Copyright (2011) Wiley–VCH Verlag GmbH & Co. KGaA.

Due to technical difficulties, it remains challenging to study metal-DNA in a metal-DNA-metal single-molecule junction system. However, taking advantage of the SWCNT junction system, Liu et al. recently reported current measurement of metallo-DNA duplex by chemically sandwiching it between two SWCNT electrodes (Figure 15) [112]. A stable metal-mediated base pair in the presence of a Cu^2+^ ion (H–Cu^2+^–H), a motif geometrically similar to the hydrogen-bonded natural base pairs, were formed at the middle of their studied DNA sequence, as shown in Figure 15b. It can also be removed after ethylenediaminetetraacetic acid (EDTA) treatment. Under a constant source-drain bias, results of the current vs. gate voltage measurements showed a sharp increase in current upon metal-base pair formation. Similar phenomena were observed when multiple metal ions were incorporated. They proposed that H–Cu^2+^–H base pairs incorporated parallel to the neighboring natural base pairs could rigidify π-stacking between DNA base pairs and mediate the electronic coupling for hole transfer, thus favoring DNA charge transport, as compared to the ligand-containing metal-free DNAs [112]. This work showed the possibility to enhance the electrical conductance of DNA by rational arrangement of multiple metal ions inside the core of the DNA base-pair stack and will encourage more future investigations toward metallo-DNA molecules bridging nanodevices.

### 4.3. DNA-Small Molecule Complex

**Figure 16:**
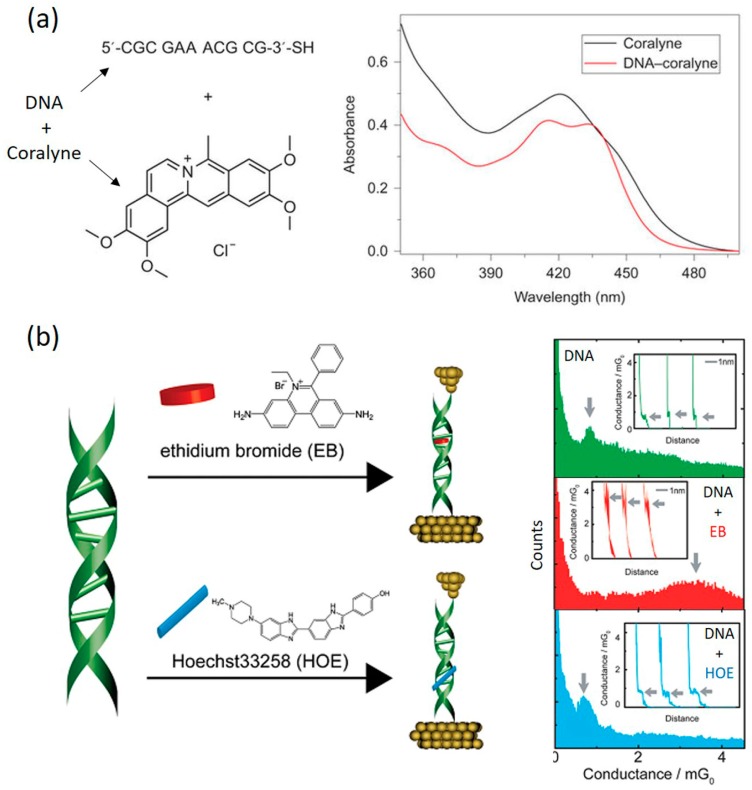
(**a**) Sequence of dsDNA, structure of coralyne molecule and their interaction revealed by UV-vis spectra. Reprinted with permission from ref. [9]. Copyright (2016) Nature Publishing Group. (**b**) Conductance measurements of dsDNA molecules with EB and HOE intercalated between the base pairs and into the grooves, respectively. Reprinted with permission from ref. [113]. Copyright (2017) Royal Society of Chemistry.

It has been shown that CT properties of π-stacked aromatic molecules can be modulated when donor or acceptor molecules were present. For example, rectification behavior was observed for the stack composed of electron donor and acceptor molecules (triphenylene and naphthalenediimide, respectively), whereas such behavior was absent in the stack composed solely of donor molecules [120]. Since π-π stacking interactions mediate the transport in both the aromatic molecule and DNA systems, the electron transport properties of DNA could thus be analogously tuned if the π-π stacking between DNA bases could be modulated. One efficient way to achieve such modulation is by intercalating small π-conjugated molecules. Experimental investigations toward this direction were recently reported. In 2016, Guo et al. [9] studied the effect of intercalating coralyne molecule (Figure 16a) to a custom-designed DNA duplex (5′-CGCGAAACGCG-3′) on its CT properties. They found that two coralyne molecules can stably intercalate into one DNA duplex, forming a DNA-coralyne complex structure which yields strong diode-like *I–V* behavior. This work will be detailed in the next section. Similarly, Harashima et al. [113] recently investigated how ethidium bromide (EB) or Hoechst 33258 (HOE), affects the electron transport of a single DNA molecule by means of the STM-BJ technique (Figure 16b). While EB has been known to intercalate between stacked DNA base pairs [121], HOE binds to the minor groove of the DNA helix and has limited effects on the helix structure [122]. They found that the conductance of DNA can be enhanced by over four folds upon EB binding due to the decreased gap between the HOMO of the DNA and the Fermi level of the electrode. It was also found that the single-molecule conductance remains almost unaffected by a groove-binding molecule, HOE, because of the absence of the interaction between the ligand and stacked bases. Results of these studies strongly suggest tuning π-π stacking of DNA bases via intercalating π-conjugated molecules as a very effective way of achieving promising functions of DNA. 

## 5. Toward DNA Molecular Diode and Transistor

**Figure 17:**
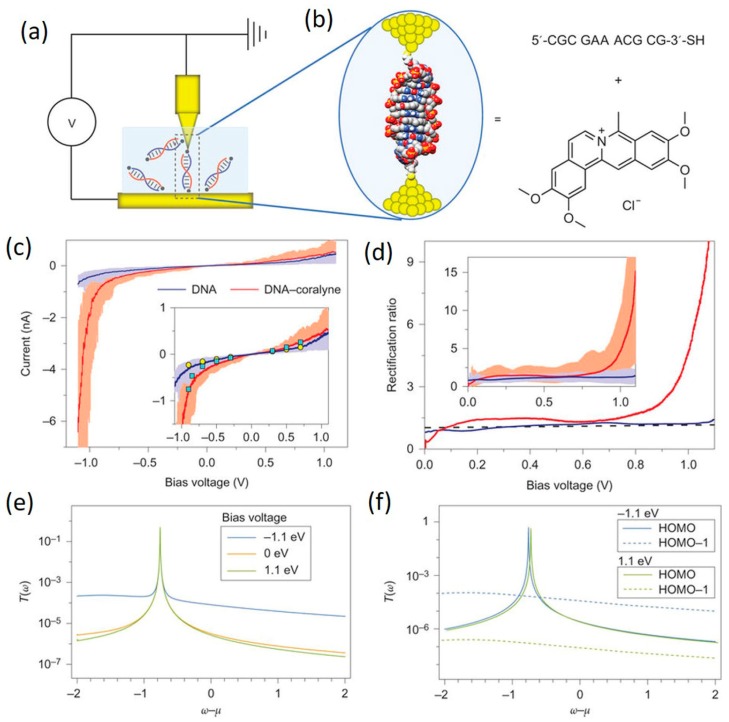
Demonstration of first DNA-based molecular diode. (**a**) STM-BJ setup; (**b**) Schematic of DNA-coralyne complex molecular junction; (**c**,**d**) show the *I–V* curves and rectification ratio curves of native DNA (blue) and DNA-coralyne complex (red); (**e**) Transmission function of DNA-coralyne complex molecular junction under different biases; (**f**) Transmission function of HOMO (solid lines) and HOMO-1 (dashed lines) levels of DNA-coralyne complex molecular junction under 1.1 V and −1.1 V. Reprinted with permission from ref. [9]. Copyright (2016) Nature Publishing Group.

The goal of molecular electronics is to functionally incorporate molecular components in electronic devices. The key to achieving this goal is to search for molecular candidates that mimic the electronic behavior of conventional semiconductors, such as diode (rectifier), which facilitates current flow in one (forward) bias direction, and transistor, current flow of which can be controlled with a gate voltage. To date, the diode-like (rectification) behavior has been observed in molecular junction devices involving small organic molecules, the structures of which usually comprise electron-donor and electron-acceptor groups [123,124,125,126]. The cause of such rectification behavior has been attributed to two main factors, asymmetric electronic structures of the molecular core [124,127] and asymmetric coupling at the two molecule-electrode contact interfaces [128,129]. For details of this topic, we refer readers to several excellent reviews (see refs. [50,127,128]). However, research using DNA as a molecular diode has been lagging behind due to poor understanding of its structure-property relation. There have been few experimental attempts until very recently. The first successful demonstration of a DNA-based molecular rectifier was reported by Guo et al. in their recent publication [9]. They created a DNA-based rectifier by intercalating two coralyne molecules into specifically designed duplex DNA molecule (5′-CGCGAAACGCG-3′) (Figure 17). The three mismatched A-A bps at the middle of the sequence facilitated the intercalation of coralyne molecules into the DNA, which turned out to significantly modulate the electronic structure of the treated DNA (Figure 17a,b). Electrical measurements of single DNA-coralyne complex molecular junctions using STM-BJ method revealed rectifying *I–V* characteristics with a high rectification of around 15 at 1.1 V (Figure 17c,d). It was noted that this is a completely counterintuitive finding considering the apparent structural symmetry of the DNA-coralyne complex. Therefore, an unprecedented transport mechanism for molecular rectification was proposed. In the new mechanism, the rectification behavior was caused by the coralyne-induced local spatial asymmetry of the distribution of electron states along the DNA chain, which results in an asymmetric change in the transmission function associated with the HOMO-1 level of the molecule. This work offered a new strategy for engineering molecular electronic elements by exploiting DNA-small molecule interaction and will ignite new interest in DNA-based functional molecular electronic devices. 

**Figure 18:**
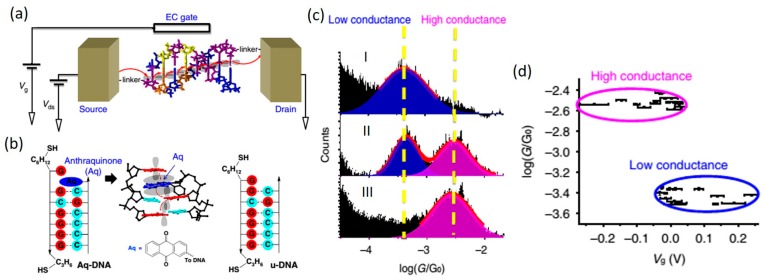
(**a**) Illustration of the STM-BJ-based electrochemical (EC) gating setup; (**b**) Redox modified DNA (Aq-DNA), where a base was replaced with an anthraquinone (Aq) moiety (highlighted in blue) at the 3′ end of a DNA strand; (**c**) Conductance histograms of Aq-DNA with the gate voltage set above (0.085 V in I), at (−0.002 V in II) and below (−0.078 V in III) the redox potential; (**d**) Conductance values at different gate voltages showing two discrete conductance states. Reprinted with permission from ref. [130]. Copyright (2017) Nature Publishing Group.

Creating a molecular field-effect transistor (FET) requires incorporating the third gating electrode into a two-terminal metal-molecule-metal junction system. A gate electrode can be fabricated aside a two-terminal molecular junction using the MCBJ and electromigration break-junction setups (EBJ) [25,50,57]. However, apart from the fact that these methods are limited in practice by low device yield, [50] measurements using these setups are often conducted in air or vacuum, which is detrimental for achieving high conductance in DNA molecules. To satisfy the requirement of aqueous environment for measurements involving DNA, STM-BJ setup proves to be ideal because it allows one to avoid leaking current between the metallic source and drain electrodes induced by counter ions in the buffer solution by coating a layer of insulating material, such as apiezon wax, onto the STM tip electrode [9,47,72]. More importantly, the promising electrochemical (EC) gating method can be incorporated into STM-BJ setup to achieve efficient gate coupling that manipulates the energy alignment and the molecular redox processes for a single-molecule junction. Using EC gating method (Figure 18a), Xiang et al. [130] recently reported their conductance measurements of a modified DNA duplex molecule where one base was replaced with a redox group for optimal EC control (Figure 18b). By applying an EC gate voltage to the molecule, they showed that switching the redox group between the oxidized and reduced states leads to reversible switching of the DNA conductance between two discrete levels (high and low) (Figure 18c,d). Their theoretical calculation shows that the conductance switching arises from a change in the molecular energy alignment associated with the redox state switching [130]. Their work successfully demonstrates the possibility of switching DNA conductance between two levels with an EC gate.

Despite of the high complexity of molecular junctions involving DNA, the unique ability of DNA to self-assemble holds huge potential for building DNA nanochips and biosensors, which is not possible with other molecules. Combined with deeper insights gained in recent years, the promising experimental results introduced in this section have paved way for more future investigations in creating functional CT out of DNA. 

## 6. Beyond Simple Charge Transport in DNA

The desire to create functional molecular devices has pushed the frontiers of both measurement capabilities and our fundamental understanding of varied physical phenomena at the single-molecule level, including mechanics, thermoelectrics, optoelectronics and spintronics in addition to electronic transport characterizations [48]. Atomic precision of single molecule devices is beyond what is achievable with many other nanomaterials. Therefore, metal-molecule-metal junction represents a powerful platform to explore a variety of physical properties beyond electron transport. This section will discuss interesting properties beyond CT achieved in DNA-based molecular junctions. 

### 6.1. DNA Spintronics 

**Figure 19:**
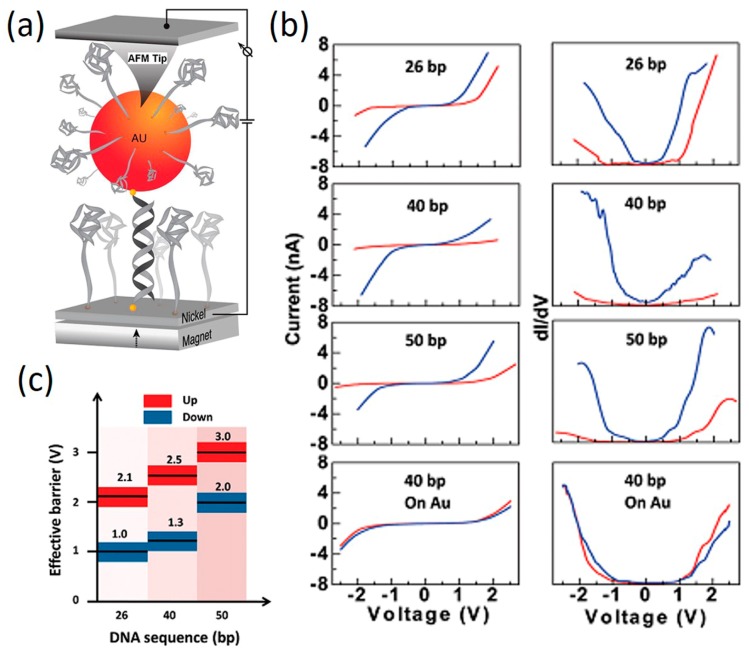
(**a**) CAFM-BJ setup for spin-dependent CT in DNA; (**b**) *I–V* curves of DNA with different lengths measured under two magnetic field polarities (up: red and down: blue); (**c**) Estimated effective barrier for DNA with different lengths. Reprinted with permission from ref. [134]. Copyright (2011) American Chemical Society.

The fact that electron can travel through chiral molecules in a spin selective manner raised the intriguing possibility that DNA CT is affected by the inherent spin of the electrons passing through it. It has been found that DNA duplex can act as a spin filter for spin selective transmission of electrons [131]. This was demonstrated in photoemission spectroscopy of DNA monolayers on Au [132] and in electrochemical charge transfer measurements on DNA monolayer films [133]. Xie et al. [134] recently reported a magnetoresistance measurement of DNA using a CAFM-BJ setup. In their work, a platinum-coated conductive AFM tip measures the current flowing from a nickel substrate through the DNA to a gold nanoparticle (Figure 19a). *I–V* characteristics were obtained for DNA duplex molecules with different lengths under two magnetic field polarities (up and down) (left column in Figure 19b). They found that the *I–V* curves are symmetrical with respect to zero bias, but *I–V* curve for Ni depends strongly on the magnetic field orientation. The magnetic field of the permanent magnet has little effect on the current flow through the DNA, but the magnetic field does affect the spin alignment in the Ni substrate. The lower traces in Figure 19b show a control experiment in which the Ni electrode was replaced by an Au electrode, which is non-ferromagnetic, and no magnetic field effect was observed. Since the dI/dV curves (right column of Figure 19b) reflect the density of states for each system qualitatively, carrier density can then be used extract an effective tunneling barrier height, as shown in Figure 19c. The difference in spin selectivity for the different DNA lengths enlarges because the effective barrier associated with the unfavorable spin is smaller for the shorter oligomers. Hence, electrons with this unfavorable spin produce higher conductance. Due to larger effective barriers for longer DNA molecules, the current associated with the unfavorable spin is blocked. 

### 6.2. DNA Piezoresistivity

**Figure 20:**
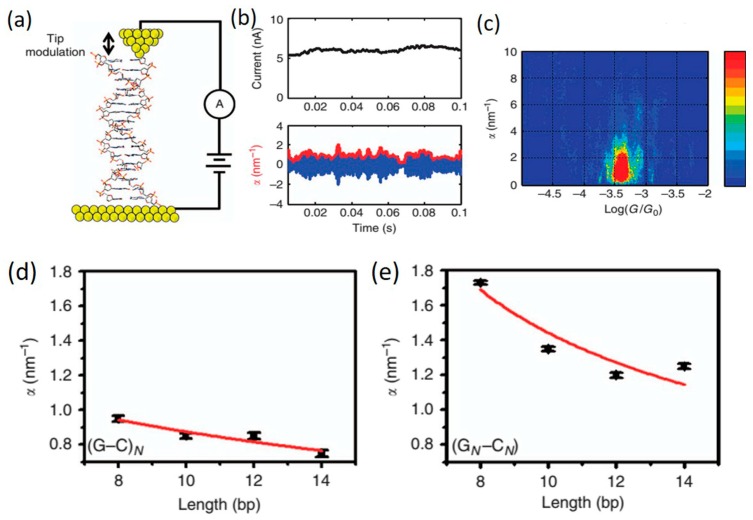
(**a**) Schematic diagram of STM-BJ with a modulating tip; (**b**) Upper: low-frequency component of the current collected from the single DNA junction; lower: the piezoresistance (α) in DNA (red curve) and conductance modulation due to tip modulation (blue curve); (**c**) α vs. conductance histogram for G-C sequence; α vs. molecular length for (**d**) (G–C)_N_ and (**e**) (G_N_–C_N_) sequences, respectively. Reprinted with permission from ref. [138]. Copyright (2015) Nature Publishing Group.

Mechanical force-induced change in the resistivity of materials is known as piezoresistivity. This important property of materials has also been observed in single molecule junction devices [135,136,137]. These behaviors arise primarily from molecule–electrode coupling effects, as opposed to distortions within the molecule causing changes of the molecular electronic states [138]. Studying piezoresistivity effect in more complex molecules such as DNA and the role that distortions of the nucleic acid units play in CT is of persistent interest. In 2015, Bruot et al. [138] demonstrated the first measurement of piezoelectricity in DNA molecular junctions (Figure 20). To measure conductance and piezoresistivity of single DNA molecules, they used a tip modulation STM-BJ technique where tip retraction was stopped and a 0.02 nm sinusoidal modulation (1 kHz frequency) was applied to the tip position along the axis of the tip when a molecular plateau was detected. To describe piezoresistance (α) in single DNA molecules, the amplitude of conductance at the modulation frequency was normalized by the molecular conductance (red curve in Figure 20b). A two-dimensional piezoresistance vs. molecular conductance histogram can be constructed (Figure 20c). They found that the piezoresistivity is larger for sequences with intra-strand purine stacking (G_N_C_N_) than for inter-strand purine sequences ((G-C)_N_). By investigating the length dependence of both piezoresistance and conductance, they also found that DNA piezoresistance is determined by the sensitive dependence of the electronic coupling between neighboring bases, along with the bridge site energy on mechanical force. Molecular orbital calculations showed that the piezoresistivity of DNA is caused by force-induced changes in the π-π electronic coupling between neighboring bases, and in the activation energy of hole hopping. 

### 6.3. DNA Thermoelectricity

**Figure 21:**
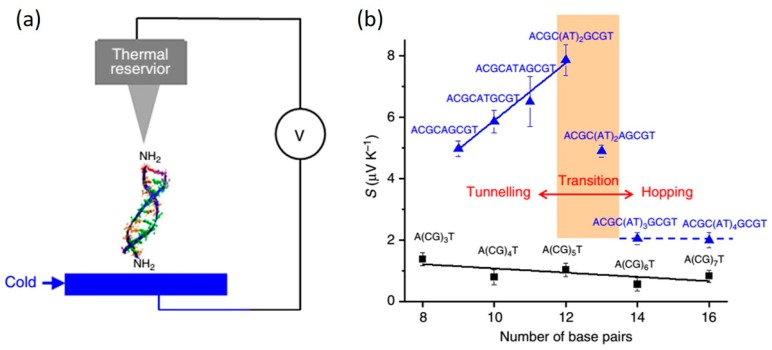
(**a**) Illustration of a DNA molecule bridged between STM tip (kept at 295 K) and substrate (cold); (**b**) The measured Seebeck coefficient (S) of DNA molecules with different sequences and lengths. Reprinted with permission from ref. [77]. Copyright (2016) Nature Publishing Group.

Thermoelectric effect, a basic property of materials, is the direct conversion of temperature difference to electric voltage and can be described by an important parameter, Seebeck coefficient. This effect in nanometer-long molecules is expected to be distinctively different from that in bulk materials [139]. The first measurement of Seebeck coefficient (S) of single molecules was reported by Reddy et al. [26]. They showed that beyond potential energy-conversion applications, the sign of Seebeck coefficient (S) of molecular junctions can indicate the nature of charge carrier (electron vs. hole) and the relative position of electrode fermi level to the frontier molecular orbitals. Stimulated by this work, increasing experimental efforts in investigating the thermoelectric effect in molecules have been devoted over the past decade. Detailed measurement techniques and the value of S for different molecules were reviewed in two recent articles (see ref. [140,141]), and will not be revisited here. Thermoelectric effect of dsDNA molecules was recently studied by Li et al. [77] using STM-BJ setup. In their experiments, the STM tip was held at 295 K while the substrate was cooled from 295 to 275 K (Figure 21a). By studying multiple sequences and lengths, they measured the S of DNA in both tunneling and hopping regime. It was found that for hopping dominant sequences (5′-A(CG)*_n_*T-3′), the value of S is small (~1 μV/K) and weakly dependent on the molecular length (black dots in Figure 21b). However, when inserting (AT)*_n_* blocks into the CG sequence, it changes both the conductance and S substantially. As shown in Figure 21b, when the number of AT block is less than four, it acts as a tunneling barrier and the S is large compared to the CG sequence and has a linear dependence in molecular length, ranging from 5 μV/K to 8 μV/K. When AT block is longer than four bps, the charge transport mechanism changes from tunneling to hopping, and S drops to smaller values (~2 μV/K) and is weakly dependent in length. For all the sequences studied, S reveals a positive sign, indicating HOMO-dominant transport. The results demonstrated in this work implies that DNA thermoelectricity may be tuned by its length and sequence.

## 7. Conclusions and Outlook

The precision control with atomic accuracy over sub-nanometer distances is the ultimate limitation of electronic device miniaturization and is unachievable with the state of the art silicon-based technologies [50]. Therefore, molecular electronics holds the greatest promise for overcoming the bottleneck of semiconductor-based technologies. The remarkable experimental and theoretical research on molecular electronics over the past two decades has exhibited promising results and significant achievement, among which DNA molecules have been playing an increasingly important role. The experiments performed on DNA-based molecular electronic devices in the past 15 years have delivered several important messages to the field that: first, short dsDNA duplex is a conductor and can be considered as a molecular wire while ssDNA and long dsDNA molecules are detrimental to facilitate CT; second, even for short DNA molecules, its CT can be dominated by completely different mechanisms depending on the specific sequence chosen, i.e., largely electron tunneling regime for AT rich sequences and hole hopping regime for GC rich sequences; third, CT through DNA is highly sensitive to its secondary structure and the surrounding ionic environment, which also means CT of DNA can be tuned by properly modulating its structure and measurement conditions; last, native dsDNA can be modified by various means, including metallization, methylation, doping, and intercalation of small molecules, which could tailor the electronic structure of DNA toward functional CT. 

In this review, we have also discussed recent experimental progress in DNA-based single-molecule electronics which suggest that due to its structural flexibility, diversity and programmability, DNA has indeed demonstrated many unique properties that are not possible in other molecules, such as sequence- and length-dependent transport, structural transition, strong interaction with metal ions and small molecules, and spin filter effect, These superior properties of DNA are believed to offer us unprecedented opportunities for the design and fabrication of molecular-scale devices and biosensors. 

However, challenges exist in several aspects. First, thorough control of DNA’s structure in different environment is necessary for both narrowing down the data distribution in CT measurements and creating long-lasting and highly stable DNA molecular devices. Second, more investigations toward functional transport properties of DNA are required both experimentally and theoretically. Special efforts should be given to improve the performance of DNA devices, such as the rectification ratio of DNA molecular rectifier and on/off ratio of DNA transistor. Third, there remains plenty of room to modify native DNA in various forms, which will require close collaboration between chemists, biologists, physics and engineers. Last, there still exists open questions in the basic field of CT in DNA. The complexity of large biomolecules like DNA makes accurate simulation of the experimental conditions difficult. More precise theoretical models and calculation algorithms that better mimic the actual experimental measurements need to be developed. 

**Figure 22:**
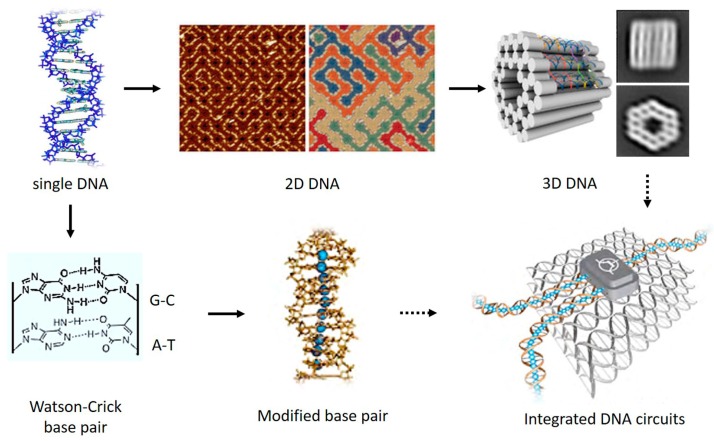
Possible routes to develop future integrated DNA circuits by combining DNA origami technologies (upper row) and appropriate modifications of the electronic structure of individual DNA molecule (lower row). The middle frame of the upper row is reprinted with permission from ref. [34]. Copyright (2017) Nature Publishing Group. The right frame of the upper row is reprinted with permission from ref. [143]. Copyright (2017) Nature Publishing Group. The middle and right frames of the lower row are reprinted with permission from ref. [116]. Copyright (2010) ELSEVIER.

Beyond single molecule transport measurements, rapid development of the emerging DNA origami technologies has been refreshing our understanding of DNA’s remarkable self-assembling ability. To date, both random shaped two-dimensional (2D) DNA nanopatterns [34,142] and three-dimensional (3D) DNA nanoblocks [143,144] can be “mass-produced” up to a scale of hundreds of nanometers in a controllable manner. There is enough reason to believe that these exciting DNA nanotechnologies will soon be applied in conjunction with single-molecule electronics to scale up DNA-based molecular circuits (Figure 22). As DNA-based molecular electronics continue to grow, one can foresee that DNA will offer a solution to many of the hurdles that need to be overcome in further scaling down electronic circuits.
